# Assessment of care pattern and outcome in hemangioblastoma

**DOI:** 10.1038/s41598-018-29047-9

**Published:** 2018-07-24

**Authors:** Yuqian Huang, Lilian Chan, Harrison X. Bai, Xuejun Li, Zishu Zhang, Yinyan Wang, Ya Cao, Giorgos Karakousis, Raymond Huang, Bo Xiao, Paul J. Zhang, Li Yang

**Affiliations:** 1 Department of Neurology, The Second Xiangya Hospital, Central South University, Changsha, Hunan 410011 China; 2 Department of Neurology, Xiangya Hospital, Central South University, Changsha, Hunan 410011 China; 3 Perelman School of Medicine, Philadelphia, Pennsylvania 19104 United States; 4 Department of Radiology, Hospital of the University of Pennsylvania, Philadelphia, Pennsylvania 19104 United States; 5 Department of Neurosurgery, Xiangya Hospital, Central South University, Hunan, 410011 China; 6 Department of Surgery, Hospital of the University of Pennsylvania, Silverstein, Philadelphia, Pennsylvania 19104 United States; 7 Cancer Research Institute, School of Basic Medicine, Central South University, Changsha, Hunan 410078 China; 8 Department of Pathology and Laboratory Medicine, Hospital of the University of Pennsylvania, Philadelphia, Pennsylvania 19104 United States; 9 Department of Radiology, The Second Xiangya Hospital, Central South University, Changsha, Hunan 410011 China; 10 Department of Radiology, Brigham and Women’s Hospital, Harvard Medical School, Boston, Massachusetts, 02120 United States; 11 Department of Neurosurgery, Beijing Tiantan Hospital, Capital Medical University, Beijing, China

## Abstract

Due to its rarity, current literature assessing prognostic factors and survival outcomes of hemangioblastoma is limited. Patients with histologically confirmed hemangioblastoma were identified from the US National Cancer Data Base. 1488 patients met inclusion criteria. 644 patients underwent gross total resection (GTR), 220 subtotal resection (STR)/biopsy, 60 stereotactic radiosurgery (SRS), 15 external beam radiotherapy (EBRT), 51 surgery followed by radiotherapy (SR + RT) and 498 no treatment. Independent predictors of shorter OS included age ≥ 40 (HR, 3.897; 95% CI, 2.341–6.487; p < 0.001), Charlson-Deyo score ≥ 1(HR, 1.756; 95% CI, 1.213–2.544; p = 0.003), tumor location in the brainstem (HR, 1.955; 95% CI, 1.129–3. 384; p = 0.017) compared to cerebellum, no treatment (HR, 2530; 95% CI, 1.533–4.177; p < 0.001) and receipt of EBRT (HR, 2.860; 95% CI, 1.073–7.618; p = 0.036) compared to STR/biopsy. GTR was associated with longer OS (HR 0.617; 95% CI, 0.391–0.974; p = 0.038), while SRS had comparable OS to STR/biopsy. The overall trend of OS by treatment modality was consistent after matching to age- and sex-matched US population data. In patients younger than 40 years, treatment was not a significant predictor of OS. In conclusion, GTR remained the optimal treatment for hemangioblastoma. SRS may perform similarly to surgery alone. Treatment was not a significant predictor of survival in younger patients.

## Introduction

Hemangioblastomas of the central nervous system (CNS) are benign vascular neoplasms that most commonly affect the cerebellum, followed by the brainstem and spinal cord^1^. The majority of CNS hemangioblastomas arise sporadically, but 20–30% of cases develop in association with Von Hippel-Lindau (VHL) disease^1^. VHL disease is an autosomal dominant, multisystem neoplastic syndrome caused by mutations in the VHL tumor suppressor gene^2^. Compared to the sporadic form, hemangioblastomas associated with VHL disease tend to manifest earlier in life and as multifocal lesions^1,3^. Long-term surveillance is required due to risk of new tumor development^4^.

Given the indolent nature of hemangioblastomas^5,6^, asymptomatic tumors may be managed with observation, and intervention is not required until symptoms develop^6^. Standard clinical management for all symptomatic presentations is complete microsurgical resection^1,7^. In general, surgical resection is a safe and effective strategy^4^. However, post-operative outcomes can vary depending on factors including disease location, number of lesions, and tumor characteristics that make complete resections difficult^8–10^. Radiotherapy (RT), in the form of stereotactic radiosurgery (SRS) or external beam radiotherapy (EBRT), can be used as primary, adjuvant, or salvage treatment strategy^11,12^. However, the current accepted practice patterns are based on small and medium -sized retrospective studies that include fewer than 200 patients, and there are no randomized clinical trials that compare these approaches^12–19^. Consequently, it is unclear if certain subgroups of patients may benefit from different management strategies.

In this study, we used the National Cancer Database (NCDB) to compare treatment strategies in patients with hemangioblastomas.

## Materials and Methods

### Study populations

The National Cancer Data Base (NCDB) is a joint project of the Commission on Cancer of the American College of Surgeons and the American Cancer Society. Established in 1989, the NCDB is a comprehensive, nationwide facility-based oncology data set that captures nearly 70% of all newly diagnosed malignancies in the United States. The data used in this study are derived from a deidentified NCDB data file. The American College of Surgeons and the Commission on Cancer have not verified and are not responsible for the analytic or statistical methodology used, or for the conclusions drawn, from these data by investigators.

**Figure 1:**
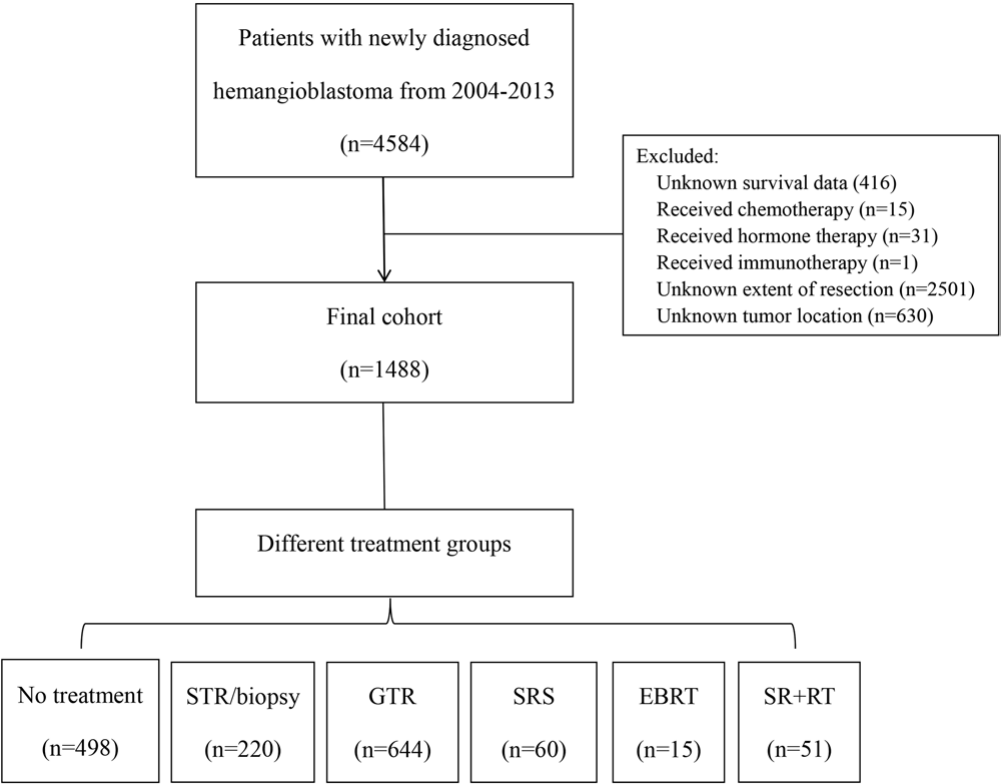
Patient selection flowchart.

The NCDB was used to identify patients with hemangioblastoma from 2004 to 2013. In this study, we excluded patients with incomplete survival data and unknown extent of resection or tumor location. The flowchart of patient selection is shown in Fig. 1. Demographic and clinical data included age, race, sex, year of diagnosis, Charlson-Deyo score, tumor size, tumor location, lesion number, metastasis, and treatments. Tumor location of ventricle, overlapping lesion of central nervous system, cauda equina and cranial nerves was categorized as “other site of CNS” per NCDB. We included patients who received both EBRT and SRS.

### Statistical methods

All data analyses were performed using SPSS 22.0 (SPSS Inc., Chicago, IL). Demographic factors and tumor characteristics of the overall cohort were analyzed using the Student’s t test for continuous variables and chi-square test for categorical variables. Univariable followed by multivariable Cox proportional hazard regression was used to identify significant predictors of OS. Variables with p value < 0.05 on univariate analysis was included in the multivariate analysis with the Cox proportional hazards model. A two-sided p value of < 0.05 was considered statistically significant. Subgroup analyses based on age were also performed. Using the R package relsurv, a “proportional excess hazard model”^20,21^ was used to analyze OS relative to age- and sex-matched population data from the Centers for Disease Control and Prevention to determine the excess risk of death associated with hemangioblastomas beyond what can be attributed to a patient’s age and sex^22^.

### Data availability

The data that support the findings of this study are available from National Cancer Database (NCDB) but restrictions apply to the availability of these data, which were used under license for the current study, and so are not publicly available. Data are however available from the authors upon reasonable request and with permission of NCDB.

## Results

### Patient characteristics

**Figure 2:**
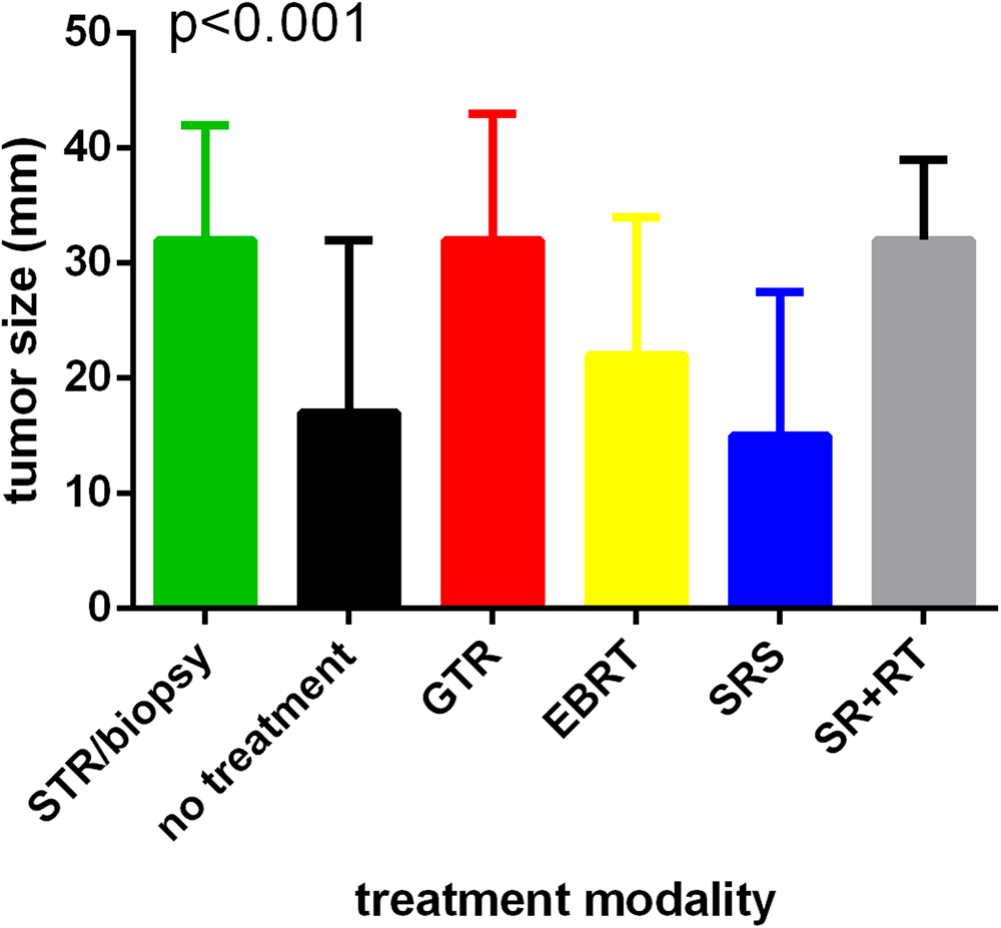
Tumor size distribution between different treatment modalities.

A total of 1488 patients met our inclusion criteria (Fig. 1). For all patients in our study, the median follow-up time was 33 months (range, 0–140 months). The median age at diagnosis was 49 years (range, 2–90 years); 55.3% patients were male; 75.6% were white; 80.6% had no significant comorbidities (Charlson-Deyo score of 0). In terms of treatment, 864 (58.2%) underwent surgery alone, 75 (5%) RT alone, 51 (3.4%) surgery followed by RT (surgery + RT), and 498 (33.5%) no treatment. Among patients who underwent surgery alone, 644 (76.9%) had gross total resection (GTR), and 220 (25.5%) subtotal resection (STR) or biopsy. Among patients who received RT alone, 60 (89%) received SRS, and 15 (20%) EBRT. Tumor size treated by SRS was much smaller than other treatments (p < 0.001; Fig. 2). The most common tumor location was the cerebellum (n = 1060, 71.2%) followed by the spinal cord (n = 145, 9.7%). A summary of demographic and clinical features of our cohort is shown in Table 1.

**Table 1:** Demographics and clinical characteristics of the overall cohort (n = 1488).

Category	Subcategory	No. (%)
Age at diagnosis, y	Median	49
	IQR	35–61
Age at diagnosis, y	<40 year	506 (34.0)
	≧40 year	982 (66.0)
Gender	Male	823 (55.3)
	Female	665 (44.7)
Race	White	1126 (75.6)
	African American	147 (9.9)
	Asian	52 (3.5)
	Hispanic	132 (8.9)
	Others/Unknown	31 (2.1)
Year of diagnosis	2004	26 (1.7)
	2005	31 (2.1)
	2006	29 (1.9)
	2007	28 (1.9)
	2008	41 (2.8)
	2009	54 (3.6)
	2010	308 (20.7)
	2011	318 (21.4)
	2012	340 (22.9)
	2013	313 (21.0)
Charlson-Deyo Score	0	1199 (80.6)
	≧1	289 (19.4)
Tumor size	<4 cm	808 (54.3)
	≧4 cm	338 (22.7)
	Unknown	342 (23.0)
Number of tumors	Unifocal	1168 (78.5)
	Multifocal	90 (6.0)
	Unknown	230 (15.5)
Tumor location	Cerebrum^+^	127 (8.5)
	Cerebellum	1060 (71.2)
	Brainstem	91 (6.1)
	Spinal cord	145 (9.7)
	Meninges	31 (2.1)
	Other site of CNS^++^	34 (2.3)
Behavior	Benign	9 (0.6)
	Borderline	1475 (99.1)
	Invasive	4 (0.3)
Metastasis	No	1382 (92.9)
	Yes	3 (0.2)
	Unknown	103 (6.9)
Treatment	No treatment	498 (33.5)
	STR/biopsy alone	220 (14.8)
	GTR alone	644 (43.3)
	SRS	60 (4.0)
	EBRT	15 (1.0)
	SR + RT	51 (3.4)
Facility location	Eastern	398 (26.7)
	Central	361 (24.3)
	Western	223 (15.0)
	Unknown	506 (34.0)
Median income	<$38,000	217 (14.6)
	$38,000–$47,999	329 (22.1)
	$48,000–$62,999	393 (26.4)
	≥$63,000	536 (36.0)
	Unknown	13 (0.9)
Proportion without high school degree in patient’s area of residence	≥21%	247 (16.6)
	13–20.9%	328 (22.0)
	7–12.9%	510 (34.4)
	<7%	392 (26.3)
	Unknown	11 (0.7)
Insurance	Uninsured	116 (7.8)
	Private insurance/managed care	866 (58.2)
	Government insurance	467 (31.4)
	Unknown	39 (2.6)
Urban/rural	Urban	1422 (95.6)
	Rural	27 (1.8)
	Unknown	39 (2.6)

Abbreviations: IQR, Inter Quartile Range; p, probability; STR, subtotal resection; GTR, gross total resection; EBRT, external beam radiotherapy; SRS, stereotactic radiosurgery; SR, surgery; RT, radiotherapy; ^+^include frontal lobe, temporal lobe, parietal lobe and occipital lobe; ^++^include ventricle, overlapping lesion of central nervous system, cauda equina and cranial nerves.

### Survival analyses

**Figure 3:**
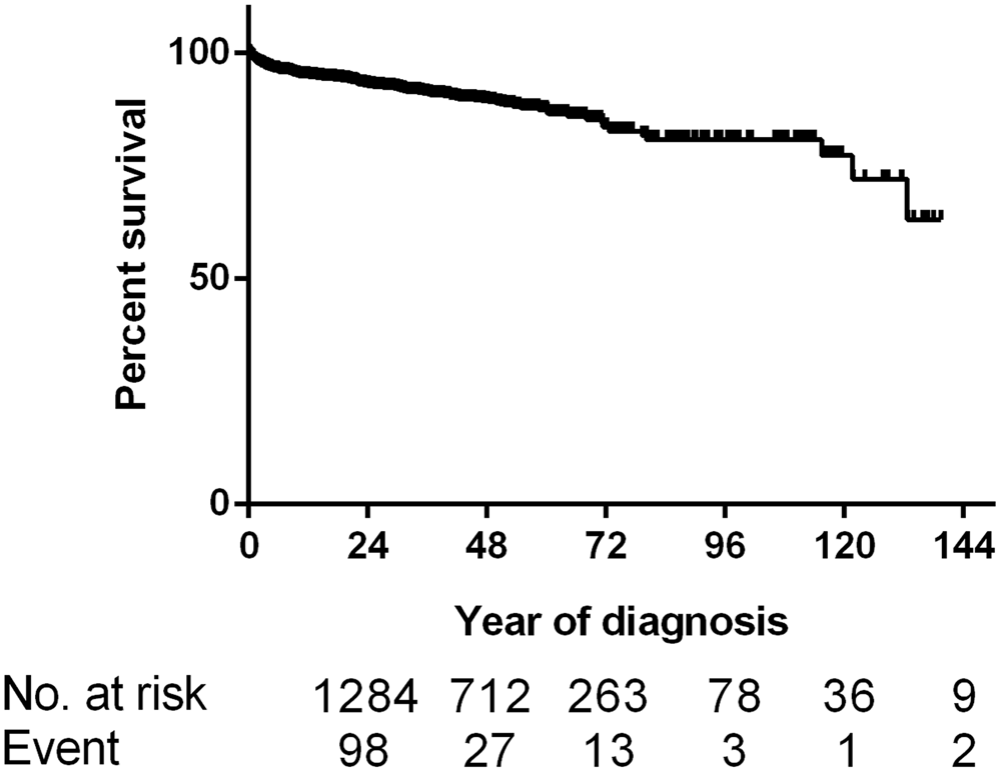
Overall survival of the entire cohort.

**Figure 4:**
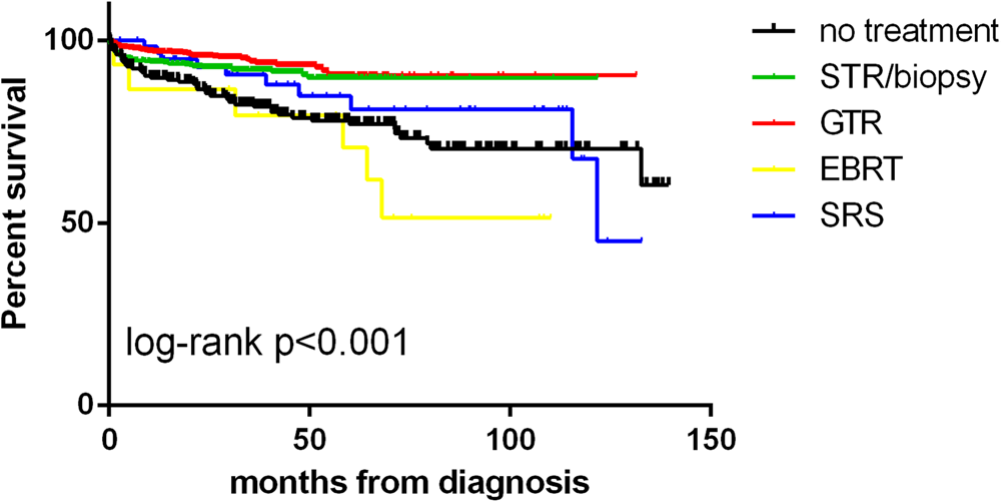
Comparison of overall survival among the different treatment groups. Supplementary Fig. 1. Overall trend of relative survival after matching to age- and sex-matched US population data.

The 1-, 3-, 5- and 10-year OS rate was 95%, 91%, 87%, 78%, respectively (Fig. 3). The 5-year OS of GTR group was 91%, followed by STR/biopsy (89%), SR + RT (88%), SRS (85%), no treatment (78%) and EBRT (71%). Independent predictors of shorter OS on multivariate Cox regression analysis included age ≥40 (HR, 3.897; 95% CI,2.341–6.487; p < 0.001), Charlson-Deyo score ≥1 (HR, 1.756; 95% CI, 1.213–2.544; p = 0.003), tumor location in the brainstem (HR, 1.955; 95% CI, 1.129–3. 384; p = 0.017) or other site of CNS (HR, 2.754; 95% CI, 1.268–5.980; p = 0.010) when compared to the cerebellum, no treatment (HR, 2.530; 95% CI, 1.533–4.177; p < 0.001) and EBRT (HR, 2.860; 95% CI, 1.073–7.618; p = 0.036) when compared to STR/biopsy. GTR was associated with longer OS (HR, 0.617; 95% CI, 0.391–0.974; p = 0.038) when compared to STR/biopsy (Fig. 4). The results of the Cox regression analyses are shown in Table 2. The overall trend of relative survival after matching to age- and sex-matched US population data was consistent, with patients who underwent GTR, SRS and surgery + RT demonstrating the best survival outcomes, followed by STR/biopsy, no treatment, and EBRT (Supplementary Fig. 1).

**Table 2:** Univariable and multivariate Cox proportional hazards analyses of overall survival (n = 1488).

Variable	Univariate analyses		Multivariate analyses	
	HR (95% CI)	P	HR (95% CI)	P
Age				
<40 years	Ref.	—	Ref.	—
≧40 years	3.919 (2.379–6.456)	<0.001*	3.897 (2.341–6.487)	<0.001*
Gender				
Male	Ref.	—		
Female	0.868 (0.622–1.210)	0.402		
Race		0.299		
White	Ref.	—		
African American	1.431 (0.868–2.360)	0.160		
Asian	1.665 (0.774–3.580)	0.192		
Hispanic	0.903 (0.485–1.680)	0.747		
Year of diagnosis				
2004–2008	Ref.	—	Ref.	—
2009–2013	0.518 (0.344–0.780)	0.002*	1.057 (0.627–1.783)	0.834
Charlson-Deyo Score				
0	Ref.	—	Ref.	—
≧1	2.006 (1.403–2.867)	<0.001*	1.756 (1.213–2.544)	0.003*
Tumor size				
<4 cm	Ref.	—		
≧4 cm	0.987 (0.653–1.491)	0.949		
Number of tumors				
Unifocal	Ref.	—		
Multifocal	0.940 (0.432–2.042)	0.875		
Tumor location		0.025*		0.019*
Cerebrum^+^	1.088 (0.595–1.989)	0.784	0.898 (0.487–1.655)	0.729
Cerebellum	Ref.	—	Ref.	—
Brainstem	2.088 (1.226–3.555)	0.007	1.955 (1.129–3. 384)	0.017
Spinal cord	1.023 (0.582–1.799)	0.936	0.781 (0.438–1.394)	0.404
Meninges	1.598 (0.647–3.949)	0.310	1.218 (0.484–3.069)	0.675
Other sites of CNS^++^	2.593 (1.201–5.598)	0.015	2.754 (1.268–5.980)	0.010
Metastasis at diagnosis				
M0	Ref.	—		
M+	0.050 (0–32455.797)	0.708		
Treatment		<0.001*		<0.001*
No treatment	2.068 (1.341–3.188)	0.001	2.530 (1.533–4.177)	<0.001
STR/biopsy alone	Ref.	—	Ref.	—
GTR alone	0.662 (0.421–1.043)	0.075	0.617 (0.391–0.974)	0.038
EBRT	3.432 (1.446–8.144)	0.005	2.860 (1.073–7.618)	0.036
SRS	1.444 (0.716–2.913)	0.305	1.459 (0.683–3.118)	0.329
SR + RT	1.242 (0.490–3.147)	0.648	1.089 (0.428–2.770)	0.858

Abbreviations: HR, Hazards Ratio; CI, confidence interval; P, probability; Ref., reference; M0, no metastasis at diagnosis; M+ , with metastasis at diagnosis; STR, subtotal resection; GTR, gross total resection; EBRT, external beam radiotherapy; SRS, stereotactic radiosurgery; SR, surgery; RT, radiotherapy; ^+^include frontal lobe, temporal lobe, parietal lobe and occipital lobe; ^++^include ventricle, overlapping lesion of central nervous system, cauda equina and cranial nerves *means statistically significant.

### Subgroup analysis based on age

Patients were divided into two groups based on an age cutoff of 40 years. The demographic and clinical features of the two age subgroups are shown in Supplementary Table 1. Younger patients were more likely to be non-white and without comorbidities, while their tumors tended to be larger, multifocal and located in the spinal cord. On multivariate Cox regression analysis, no treatment (adjusted HR, 2.578; 95% CI, 1.528–4.351; p < 0.001) and EBRT (adjusted HR, 4.199; 95% CI, 1.573–11.207; p = 0.004) were associated with shorter OS in older patients, while GTR was associated with longer OS when compared to biopsy/STR (adjusted HR, 0.571; 95% CI, 0.349–0.935; p = 0.026). In contrast, treatment was not a significant predictor of OS for younger patients. The results of the Cox regression analyses are shown in Supplementary Tables 2 and 3.

## Discussion

Due to its rarity, current literature assessing prognostic factors and survival outcomes of hemangioblastoma is limited^9,17,23–25^. Consequently, the established guidelines for treatment are based on small to medium-sized retrospective studies. In this study, we analyzed a large cohort of patients with hemangioblastomas using the NCDB.

Our analysis found that receipt of surgery was associated with longer OS. Due to the stuttering growth pattern and indolent nature of hemangioblastomas, symptomatic progression of these tumors is difficult to predict^6,26^. Therefore, while asymptomatic sporadic tumors can be managed with observation, therapeutic intervention is generally recommended. This is supported in our analysis with receipt of surgery having better outcomes than no treatment. Tumor size was not associated with survival on univariate analysis. However, patients with larger tumors were more likely to undergo resection. Notably, due to small cohort sizes, there is very little data in the current literature assessing survival outcomes based on extent of resection. However, several studies have reported increased rate of intra-operative and post-operative hemorrhage in patients who underwent a partial resection, likely secondary to the highly vascular nature of the tumor^4,7,17,27–29^. This suggests that STR may carry a greater risk of morbidity.

Our results showed that GTR resulted in longer OS than STR/biopsy. Nevertheless, cases in which aggressive tumor removal may be detrimental for patient outcomes should always be considered, as GTR has the potential to damage nearby cranial structures and produce significant patient morbidity. This may be particularly relevant for hemangioblastomas originating from the brainstem as these tumors were found to have worse OS when compared to cerebellar tumors in our cohort and are oftentimes proximal to critical structures. A number of studies have emphasized the need for meticulous resection of brainstem lesions for this reason^8,23,29,30^.

Compared to resection, the role of RT in the treatment of hemangioblastomas is less well-defined. Given the considerable interest in the use of RT as a minimally invasive primary or adjuvant treatment strategy^13,18,31^, we analyzed how primary SRS and EBRT perform compared to surgery in this cohort. Our results demonstrate that patients who underwent SRS or EBRT as primary treatment had smaller tumor size when compared to patients who underwent surgery, while size of tumor was similar to surgery alone in patients who had adjuvant RT after surgery. On relative survival analysis, SRS trended towards a similar relative survival compared to GTR. In contrast, on Cox regression analysis, SRS yielded comparable survival outcomes to STR/biopsy. These results are limited by the small number of patients undergoing primary SRS in our cohort, but may indicate that SRS at least provides comparable survival outcomes to surgery. In the literature, while several studies have presented outcomes for hemangioblastomas treated by SRS^11,18,19,24,32–34^, none has directly compared their findings to resection. In a study of 186 patients, Kano *et al*. found that SRS provided decent tumor control with improved outcomes for small, solid-type tumors but was unable to compare those results to surgical outcomes^11^. While limited by our cohort size, our findings support the use of SRS as a reasonable alternative to surgery alone especially for small tumors. Additionally, EBRT is generally only indicated when surgery and SRS are not viable options^12^. Studies describing the use of EBRT in the management of hemangioblastoma are very limited. In the largest paper discussing use of EBRT (n = 18), Koh *et al*. noted this modality has a role in managing extensive, multifocal disease, tumors adjacent to critical structures, and treatment of residual or recurrent tumors^12^. In our study, receipt of EBRT was associated with a significantly worse OS, and this may be related to the unfavorable prognostic factors in patients who were selected for this treatment. Nevertheless, given the limited number of patients in our cohort undergoing SRS (n = 60) and EBRT (n = 15) as primary treatment, further investigation is necessary to fully evaluate the role of RT in management of this disease. Although we did not find a survival benefit of post-operative RT, the small number of patients undergoing both surgery and post-operative RT in our cohort, (n = 51) made it difficult to assess the efficacy of RT as an adjuvant treatment.

When our cohort was stratified by age, we found that for patients <40 years of age, treatment was not a significant prognostic factor of survival. In contrast, treatment remained a predictive factor for patients >40 years of age, with similar trend to the overall cohort. These findings may be related to the fact that younger patients are more likely to have VHL-related hemangioblastomas, and older patients are more likely to have sporadic tumors. The average age at time of diagnosis for VHL patients is generally in the third decade^1,5,6,9,35^ with one series noting all VHL-associated hemangioblastoma cases presenting at age 40 or younger^36^. In contrast, average age at time of diagnosis for sporadic cases is much more likely to be greater than 40 years^1,9,35^. Higher prevalence of brainstem and multifocal lesions in the <40 age group also suggests that the younger age group encompasses the majority of patients with VHL disease in our cohort. What remains a significant challenge for providers is determining the optimal management strategy for VHL patients in which disease progression is attributed to manifestations of symptoms associated with new tumor development. The frequency of intervention should be minimized in order to avoid additional treatment-related morbidities over time^4,5^. Our findings underscore the need for careful longitudinal surveillance of VHL patients because successful therapy may not lead to better survival outcomes.

We acknowledge a few limitations. First, there was selection bias associated with a retrospective analysis. Second, because there was no central pathology review, misdiagnosed cases of hemangioblastomas could be present in our study cohort. Third, because data on recurrence was not available, progression-free survival could not be assessed. Likewise, data on clinical manifestations or neurological status was not provided by the NCDB, limiting the conclusions that can be made about site-specific surgical risks and outcomes^37^. Fourth, the limited size and quality of data makes it difficult to draw definite conclusion on the efficacy of RT. Finally, the database does not have information on VHL, so we used age <40 as a surrogate marker for a VHL diagnosis. Despite these limitations, the large sample size allows for meaningful trends to be observed with adequate power across multiple healthcare systems. Further studies focusing on patients with a definite VHL diagnosis and those who were treated with RT are needed to guide management of these specific populations.

## Conclusion

Patients with smaller tumors were more likely to undergo no treatment or treated by SRS or EBRT as primary treatment. Brainstem tumors had worse outcomes than cerebellar tumors. GTR remained the optimal treatment for hemangioblastoma. SRS as primary treatment may perform similarly to surgery alone. Treatment was not a significant predictor of survival in younger patients with hemangioblastoma.

## Electronic supplementary material

supplementary information
