# Supplementary material for: Assessment of care pattern and outcome in hemangioblastoma
Source: Sci Rep. 2018 Jul 24;8:11144. doi: 10.1038/s41598-018-29047-9 (PMC6057968; doi:10.1038/s41598-018-29047-9)

Title Page

Title: Assessment of care pattern and outcome in hemangioblastoma

Authors:

1. Yuqian Huang[1]*
2. Lilian Chan[3]*
3. Harrison X. Bai[4]
4. Xuejun Li[5]
5. Zishu Zhang[9]
6. Yinyan Wang[11]
7. Ya Cao[7]
8. Giorgos Karakousis[6]
9. Raymond Huang[10]
10. Bo Xiao[2]
11. Paul J. Zhang[8]
12. Li Yang[1]

Affiliations:

1. Department of Neurology, The Second Xiangya Hospital, Central South University, Changsha, Hunan, China, 410011.
2. Department of Neurology, Xiangya Hospital, Central South University, Changsha, Hunan, China, 410011.
3. Perelman School of Medicine, Philadelphia, Pennsylvania, United States, 19104.
4. Department of Radiology, Hospital of the University of Pennsylvania, Philadelphia, Pennsylvania, United States, 19104.
5. Department of Neurosurgery, Xiangya Hospital, Central South University, Hunan, China, 410011.
6. Department of Surgery, Hospital of the University of Pennsylvania, Silverstein, Philadelphia, Pennsylvania, United States, 19104.
7. Cancer Research Institute, School of Basic Medicine, Central South University, Changsha, Hunan, China, 410078.
8. Department of Pathology and Laboratory Medicine, Hospital of the University of Pennsylvania, Philadelphia, Pennsylvania, United States, 19104.
9. Department of Radiology, The Second Xiangya Hospital, Central South University, Changsha, Hunan, China, 410011.
10. Department of Radiology, Brigham and Women's Hospital, Harvard Medical School, Boston, Massachusetts, United States, 02120.
11. Department of Neurosurgery, Beijing Tiantan Hospital, Capital Medical University, Beijing, China

Footnote:*These two authors contributed equally to this paper.

Corresponding Author:

1. Dr. Li Yang
   Address: Department of Neurology, The Second Xiangya Hospital, Central South University, No.139 Middle Renmin Road, Changsha, Hunan, 410011, China.
   E-mail address: yangli762@csu.edu.cn
   Phone: +8615116291599
   Fax: +86073185295856

2. Dr. Bo Xiao
   Address: Department of Neurology, Xiangya Hospital, Central South University, Changsha, Hunan, China, 410011.
   E-mail address: Xiaobo_xy@126.com
   Phone: 13908487631

Supplementary Table 1. Comparison of demographic and clinical characteristics between younger and older patients.

Category		No. (%)		p
		**Patients <40 y n=506**	**Patients ≧40 y n=982**	
Gender	Male	267 (52.8)	556 (56.6)	0.157
	Female	239 (47.2)	426 (43.4)	
Race	White	338 (66.8)	788 (80.3)	<0.001*
	African American	68 (13.4)	79 (8.0)	
	Asian	21 (4.2)	31 (3.2)	
	Hispanic	65 (12.8)	67 (6.8)	
	Others/Unknown	14 (2.8)	17 (1.7)	
Year of diagnosis	2004	13 (2.6)	13 (1.3)	0.403
	2005	10 (2.0)	21 (2.1)	
	2006	7 (1.4)	22 (2.2)	
	2007	10 (2.0)	18 (1.8)	
	2008	14 (2.8)	27 (2.7)	
	2009	24 (4.7)	30 (3.1)	
	2010	95 (18.8)	213 (21.7)	
	2011	103 (20.4)	215 (21.9)	
	2012	123 (24.3)	217 (22.1)	
	2013	107 (21.0)	206 (21.1)	
Charlson-Deyo Score	0	454 (89.7)	745 (75.9)	<0.001*
	≧1	52 (10.3)	237 (24.1)	
Tumor size	< 4 cm	251 (49.6)	557 (56.7)	0.029*
	≧4 cm	130 (25.7)	208 (21.2)	
	Unknown	125 (24.7)	217 (22.1)	
Number of tumors	Unifocal	367 (72.5)	801 (81.5)	<0.001*
	Multifocal	56 (11.1)	34 (3.5)	
	Unknown	83 (16.4)	147 (15.0)	
Tumor location	Cerebrum[+]	38 (7.5)	89 (9.1)	0.033*
	Cerebellum	342 (67.5)	718 (73.1)	
	Brainstem	38 (7.5)	53 (5.4)	
	Spinal cord	63 (12.5)	82 (8.4)	
	Meninges	10 (2.0)	21 (2.1)	
	Other site of CNS[++]	15 (3.0)	19 (1.9)	
Behavior	Benign	4 (0.8)	5 (0.5)	0.747
	Borderline	501 (99.0)	974 (99.2)	

	Invasive	1 (0.2)	3 (0.3)	
Metastasis at diagnosis	No	464 (91.7)	918 (93.5)	0.271
	Yes	2 (0.4)	1 (0.1)	
	Unknown	40 (7.9)	63 (6.4)	
Treatment	No treatment	90 (17.8)	130 (13.2)	0.097
	STR/biopsy alone	176 (34.8)	322 (32.8)	
	GTR alone	196 (38.7)	448 (45.6)	
	SRS	22 (4.3)	11 (1.1)	
	EBRT	4 (0.8)	38 (3.9)	
	Surgery and radiotherapy	18 (3.6)	33 (3.4)	
Facility location	Eastern	0	398 (40.5)	/
	Central	0	361 (36.8)	
	Western	0	223 (22.7)	
	Unknown	506 (100)	0	
Median income	<$38,000	88 (17.4)	129 (13.1)	0.112
	$38,000-$47,999	111 (21.9)	218 (22.2)	
	$48,000-$62,999	138 (27.3)	255 (26.0)	
	≥$63,000	163 (32.2)	373 (38.0)	
	Unknown	6 (1.2)	7 (0.7)	
Proportion without high school degree in patient's area of residence	≥21%	107 (21.1)	140 (14.3)	0.037*
	13%-20.9%	108 (21.3)	220 (22.4)	
	7%-12.9%	163 (32.3)	347 (35.3)	
	<7%	122 (24.1)	270 (27.5)	
	Unknown	6 (1.2)	5 (0.5)	
Insurance	Uninsured	63 (12.5)	53 (5.4)	<0.001*
	Private insurance/managed care	327 (64.5)	539 (54.9)	
	Government insurance	95 (18.8)	372 (37.9)	
	Unknown	21 (4.2)	18 (1.8)	
Urban/rural	Urban	478 (94.5)	944 (96.2)	0.709
	Rural	10 (2.0)	17 (1.7)	
	Unknown	18 (3.5)	21 (2.1)	

Abbreviations: P, probability; M0, no metastasis at diagnosis; M+, with metastasis at diagnosis; STR, subtotal resection; GTR, gross total resection; EBRT, external beam radiotherapy; SRS, stereotactic radiosurgery; +, include frontal lobe, temporal lobe, parietal lobe and occipital lobe; ++, include ventricle and overlapping lesion of brain.

Supplementary Table 2. Univariable and multivariate Cox proportional hazards analyses of overall survival in patients ≥ 40.

Variable	Univariate analyses		Multivariate analyses	
	HR (95% CI)	P	HR (95% CI)	P
Gender				
Male	Ref.	-		
Female	0.901 (0.631-1.288)	0.569		
Race		0.525		
White	Ref.	-		
African American	1.457 (0.817-2.601)	0.203		
Asian	1.491 (0.606-3.671)	0.385		
Hispanic	1.018 (0.495-2.094)	0.962		
Year of diagnosis				
2004-2008	Ref.	-	Ref.	-
2009-2013	0.526 (0.340-0.814)	0.004*	1.276 (0.736-2.212)	0.385
Charlson-Deyo Score				
0	Ref.	-		
≧1	1.391 (0.946-2.046)	0.093		
Tumor size				
< 4 cm	Ref.	-		
≧4 cm	1.181 (0.763-1.829)	0.455		
Number of tumors				
Unifocal	Ref.	-		
Multifocal	1.631 (0.684-3.889)	0.270		
Tumor location		0.266		
Cerebrum+	0.931 (0.483-1.794)	0.830		
Cerebellum	Ref.	-		
Brainstem	1.568 (0.814-3.023)	0.179		
Spinal cord	1.104 (0.601-2.026)	0.750		
Meninges	1.385 (0.556-3.449)	0.485		
Other sites of CNS++	2.661 (1.079-6.564)	0.034		
Treatment		<0.001*		<0.001*
No treatment	2.298 (1.446-3.650)	<0.001	2.578 (1.528-4.351)	<0.001
STR/biopsy alone	Ref.	-	Ref.	-
GTR alone	0.567 (0.346-0.927)	0.024	0.571 (0.349-0.934)	0.026
EBRT	3.439 (1.433-8.250)	0.006	4.199 (1.573-11.207)	0.004
SRS	1.670 (0.818-3.408)	0.159	1.935 (0.886-4.228)	0.098
SR+RT	1.383 (0.542-3.531)	0.498	1.381 (0.541-3.527)	0.499

Abbreviations: HR, Hazards Ratio; CI, confidence interval; P, probability; Ref., reference; STR, subtotal resection; GTR, gross total resection; EBRT, external beam radiotherapy; SRS, stereotactic radiosurgery; +, include frontal lobe, temporal lobe, parietal lobe and occipital lobe; ++, include ventricle and overlapping lesion of brain.

Supplementary Table 3. Univariable and multivariate Cox proportional hazards analyses of overall survival in patients < 40.

Variable	Univariate analyses		Multivariate analyses	
	HR (95% CI)	P	HR (95% CI)	P
Gender				
Male	Ref.	-		
Female	0.895 (0.353-2.269)	0.814		
Race		0.071		
White	Ref.	-		
African American	3.638 (1.176-11.251)	0.025		
Asian	4.891 (1.023-23.379)	0.047		
Hispanic	1.876 (0.494-7.132)	0.356		
Year of diagnosis				
2004-2008	Ref.	-		
2009-2013	0.437 (0.137-1.399)	0.163		
Charlson-Deyo Score				
0	Ref.	-	Ref.	-
≧1	6.993 (2.638-18.541)	<0.001*	5.879 (2.122-16.289)	0.001*
Tumor size				
< 4 cm	Ref.	-		
≧4 cm	0.560 (0.156-2.013)	0.375		
Number of tumors				
Unifocal	Ref.	-		
Multifocal	0.575 (0.074-4.453)	0.596		
Tumor location		0.005*		0.019*
Cerebrum+	2.873 (0.578-14.277)	0.197	3.275 (0.654-16.399)	0.149
Cerebellum	Ref.	-	Ref.	-
Brainstem	9.063 (2.911-28.216)	<0.001	6.306 (1.959-20.300)	0.002
Spinal cord	1.751 (0.352-8.713)	0.494	1.442 (0.287-7.240)	0.656
Other sites of CNS++	7.928 (1.594-39.438)	0.011	8.633 (1.730-43.080)	0.009
Treatment		0.959		
No treatment	1.827 (0.530-6.293)	0.340		
STR/biopsy alone	Ref.	-		
GTR alone	1.116 (0.340-3.657)	0.856		

Abbreviations: HR, Hazards Ratio; CI, confidence interval; P, probability; Ref., reference; STR, subtotal resection; GTR, gross total resection; +, include frontal lobe, temporal lobe, parietal lobe and occipital lobe; ++, include ventricle and overlapping lesion of brain.

Supplementary Figure 1. Overall trend of relative survival after matching to age- and sex-matched US population data.

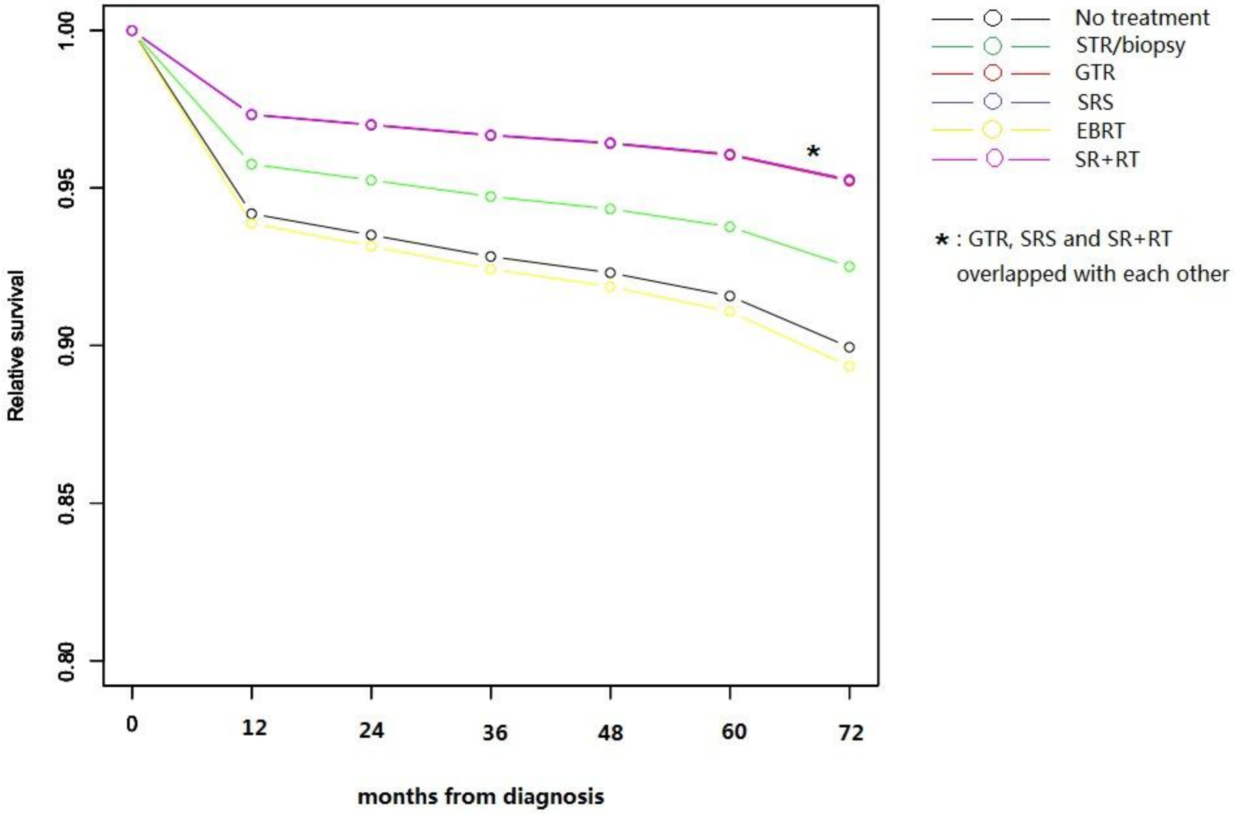

Supplement: Supplementary file 1 — supplementary information [file 41598_2018_29047_MOESM1_ESM.pdf]
